# Reinforced technique of tension band wiring for tibial tuberosity fracture in adolescents: a case report for surgical technique

**DOI:** 10.1097/MS9.0000000000000795

**Published:** 2023-05-10

**Authors:** Yong-Geun Park, Seung J. Yoo, Yong-Beom Kim, Youjeong Jang, Chaemoon Lim

**Affiliations:** a Department of Orthopaedic Surgery, Jeju National University Hospital; b Department of Orthopedic Surgery, Soon Chun Hyang University Seoul Hospital, Seoul, South Korea; c School of Medicine, Jeju National University, Jeju, South Korea

**Keywords:** adolescent, reinforced technique, tension band wiring, tibial tuberosity fracture

## Abstract

Most of the fractures had been previously treated with open reduction and internal fixation. Although there are a number of fixation methods, a consensus on the treatment options has not been reached yet.

**Case presentation:**

We demonstrated the reinforced technique of tension band wiring and its surgical outcomes in the tibial tuberosity fracture of a 14-year-old male basketball player. For the modified technique, the wire was inserted between the patellar tendon and tibial tuberosity (insertion site of patellar tendon) and passed distally through the 2-mm-sized predrill cortical hole. Tightening the figure of the eight loops draws the fractured fragments together and anatomically reduces under appropriate compression. This technique can achieve the reduction and fixation of the fracture simultaneously. We confirmed the fixation stability with a range of knee joint motions. The patient was able to return back to the pre-injury level of sports activity at postoperative 2 months.

**Clinical discussion:**

The original technique of tension band wiring utilized the Kirschner wire to make a figure-of-eight loop. However, we used the patellar tendon and its insertion site of the tibial tuberosity for making a figure-of-eight loop. Moreover, the reduction and fixation of fracture were achieved simultaneously by tightening the tension band wire. This reinforced technique was firm enough for postoperative rehabilitation.

**Conclusion:**

The most certain advantage of this technique was to be able to reduce anatomically and fixate firmly with appropriate compression simultaneously. We recommend open reduction internal fixation with the reinforced technique of tension band wiring for displaced tibial tuberosity fracture in adolescent athletes.

## Introduction

Highlights

- Although there are a number of fixation methods for tibial tuberosity fracture, a consensus on the treatment options has not been reached yet.
- In the case report, we reinforced the tendon band wiring technique for tibial tuberosity fracture.
- With this reinforced technique of tendon band wiring, the reduction and fixation of tibial tuberosity fracture were achieved simultaneously by tightening the tension band wire.
- Moreover, the rigid fixation and early rehabilitation allow young athletes to resume their previous sports activity as soon as possible.

Tibial tuberosity fractures account for 3% of tibial fractures and less than 1% of physeal plate injuries^1,2^. The injury mainly occurs in athletic males between 14 and 17 years of age as the tibial physis begins to fuse^3^. The fracture usually results from the strong concentric contraction of the quadriceps with the knee in extension during jumping or powerful eccentric contraction of the quadriceps with the knee in flexion during landing from a jump^4,5^. When the tensile forces exerted by the quadriceps are transmitted to the anterior tibial tuberosity through the patellar tendon, the tibial tuberosity fracture may occur^6^.

There are some concerns about the potential arrest of the growth plate and the development of arthritic changes after tibial tuberosity fracture because the fracture involves the growth plate and articular surface. The goal of treatment for tibial tuberosity fracture is to restore the extensor mechanism of the knee and the articular surface^3^. Furthermore, the early return of the previous activity level is another goal since fracture usually occurs in adolescent athletes^7^. Conservative treatment with cast immobilization of the knee in extension can be attempted for fractures with minimal displacement (<2 mm)^8^. However, most of the fractures were treated with surgical management with open reduction and internal fixation. Although there are many fixation methods, including cannulated screws, Kirshner wires, suture anchors, tension band wiring, or a combination of these, a consensus on the treatment options has not been reached yet^9^.

In this case report, we described the reinforced technique of tension band wiring and its surgical outcomes for tibial tuberosity fracture in adolescent athletes. This case report has been performed in line with the Surgical Case Report (SCARE) criteria^10^.

## Case report

**Figure 1:**
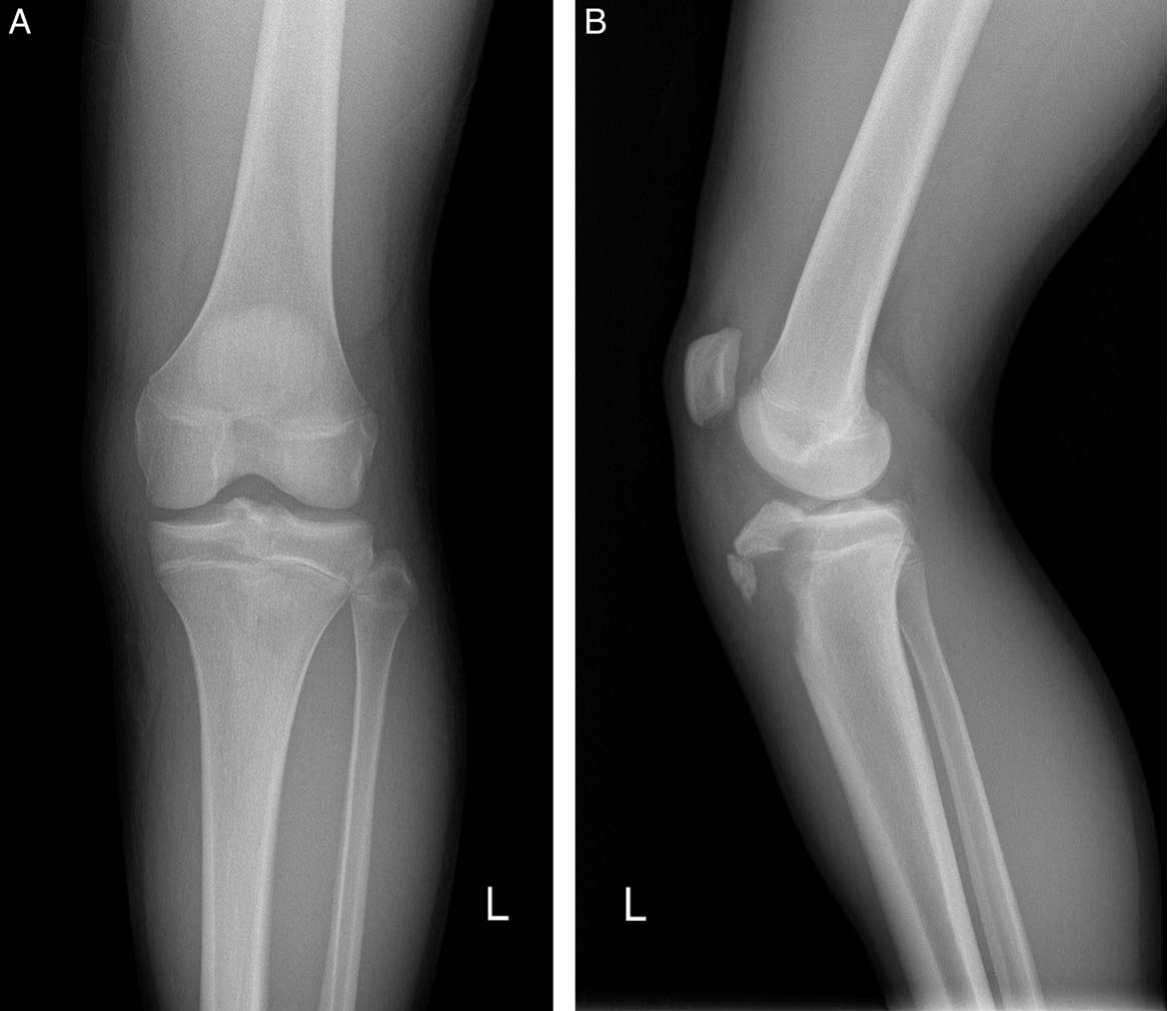
Anteroposterior (A) and lateral (B) plain radiographs showed an Ogden classification type III fracture of the tibial tuberosity in a 14-year-old.

A 14-year-old male basketball player with no specific medical history was referred to our hospital with acute pain in the left knee. The pain occurred when he took off after jumping while playing basketball. Physical examination revealed swelling and tenderness on the proximal tibia area. He was unable to extend the knee. The plain radiograph showed a displaced, multifragmentary tibial tuberosity fracture involving the proximal tibial articular surface (Ogden classification type III) (Fig. 1).

**Figure 2:**
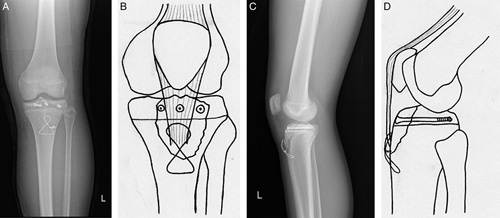
Anteroposterior (A) and lateral (C) plain radiographs showed the fracture was treated by internal fixation with tension band wiring supplemented by a cannulated screw. (B, D) Scheme of this content.

Two days after admission, under general anesthesia, open reduction and internal fixation were performed in a supine position by a senior pediatric orthopedic surgeon. After a midline skin incision was made, the fracture site was carefully cleared of debris and hematoma. For the reinforced technique of tension band wiring, we identified the tibial tuberosity and its insertion site for the patellar tendon and prepared a 2-mm-sized drill hole in the proximal tibial diaphysis at 2 cm distal to the fracture site. The wire (1.5 mm, 16 gauge stainless steel wire) was inserted between the patellar tendon and tibial tuberosity (insertion site of patellar tendon). Then, the wire wrapped proximally around the patellar tendon at its insertion site on the fractured fragment and passed distally through the 2-mm-sized predrill cortical hole in the figure-of-eight configurations. Tightening the figure of the eight loops draws the fractured fragments together and anatomically reduces under appropriate compression. This reinforced technique of tension band wiring wrapping around the patellar tendon can achieve the reduction and fixation of the fracture simultaneously. We confirmed the fixation stability with a range of knee joint motions. Internal fixation was supplemented by 4.0 mm cannulated at proximal tibia epiphysis (Fig. 2). The torn periosteum and patellar tendon were repaired with a suture anchor.

**Figure 3:**
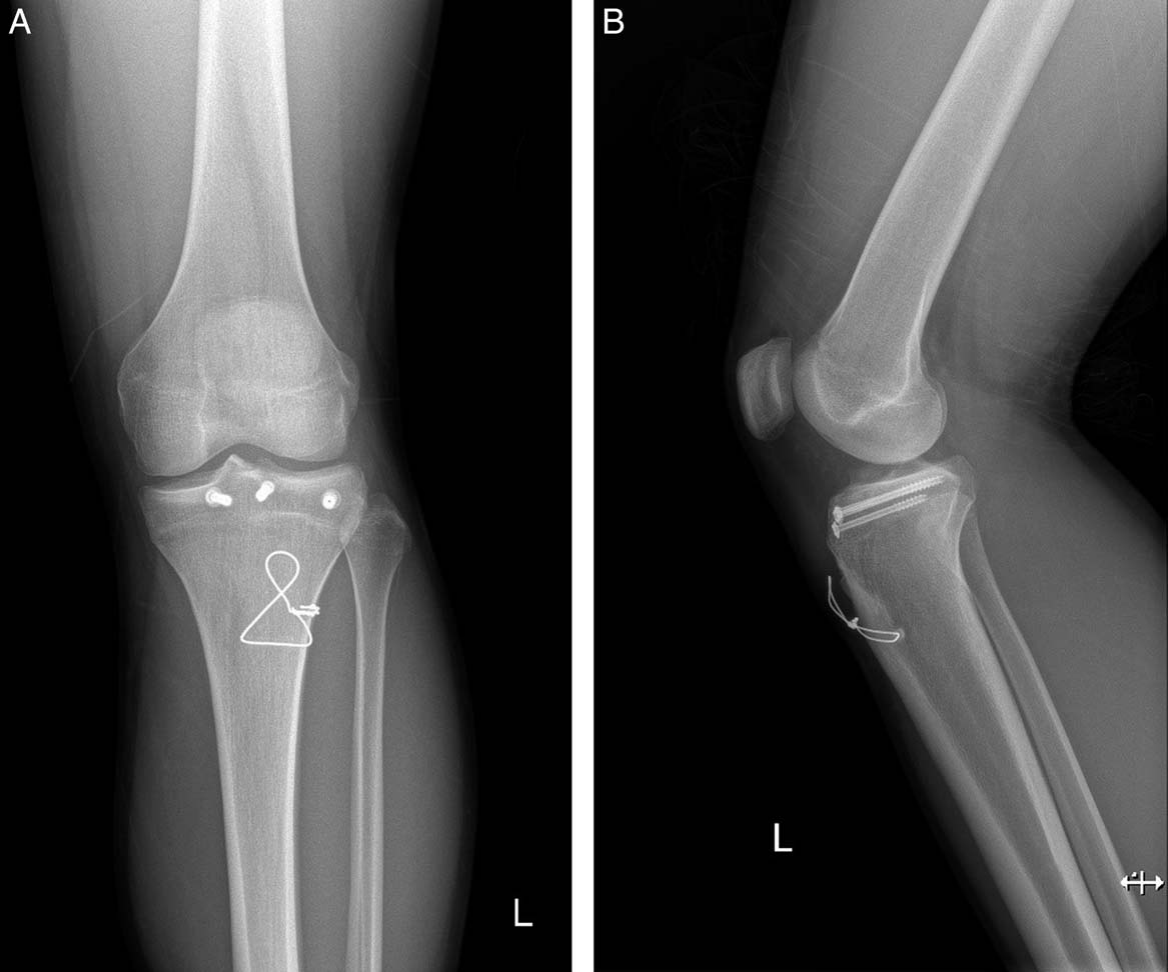
Two years after the operation, anteroposterior (A) and lateral (B) plain radiographs showed the growth plate was closed, and no complications were observed.

The knee was immobilized in a long leg cast at 5° of knee flexion, which was applied postoperatively. After 2 weeks, active and passive knee range of motion was started. At the postoperative 6th week, a full range of motion was achieved without any pain, and the extension power of the operated knee was equal to that of the contralateral side. The patient was able to return to the pre-injury level of sports activity after 2 months postoperatively. During the yearly outpatient follow-up at the end of the postoperative 2nd year, the patient did not complain of any wire-related pain or any other complications (Fig. 3).

## Discussion

The treatment of tibial tuberosity fracture primarily relies on the fracture pattern and injury severity. The primary goal of treatment is to restore the extensor mechanism, the articular surface, and the meniscal anatomy, and various fixation methods, such as cannulated screws, Kirshner wires, suture anchors, tension bands, or a combination of these, can be used^9^. In this case, we preferred fixation with tension band wiring supplemented by cannulated screw. With the tension band wiring technique, the reduction and fixation of fracture can be achieved spontaneously by tightening the figure of the eight loops. The tension band wiring neutralizes the tensile force of the fracture site and compresses the fractured apophysis to its bed^7^. We thought this reinforced technique of tension band wiring would allow easier reduction and rigid fixation for tibial tuberosity fracture.

There are two options for the treatment of tibial tuberosity fracture: conservative treatment and operative treatment. Non-displaced or minimally displaced (<2 mm, Ogden classification type I or II) can be treated with a long leg cast in extension for 3–4 weeks. However, most of the displaced fractures are treated with surgical management via open reduction and internal fixation to restore the extensor mechanism of the knee and the joint surface^3^. Pretell-Mazzini *et al*. reported that most (88%) of the tibial tuberosity fractures were treated with surgical management, and open reduction (98%) was used rather than closed reduction in their systematic review. Despite a number of fixation methods with various fixation materials, there has not yet been a consensus on surgical management and its fixation methods^9^.

Among various fixation methods, cannulated screws or tension band wiring are frequently used. Ares *et al*. reported that open reduction and internal fixation with screws placed parallel to the joint surface above and below the physis were safe and provided a stable fixation that allowed the patients to resume sports at the same level as prior to the injury. They did not apply tension band wiring because the screw fixation was strong enough, and a cast was applied postoperatively^11^. Nikiforidis *et al*. introduced the fracture fixation achieved by one cancellous screw and supplemented by tension band wiring. The wire was driven proximally around the superior pole of the patella and distally around the upper tibial diaphysis. They did not apply a cast and the immediate rehabilitation was started on the first postoperative day^9^. In the current case, we modified the tendon band wiring technique. The original technique of tension band wiring utilized the Kirschner wire to make a figure-of-eight loop. However, we used the patellar tendon and its insertion site of the tibial tuberosity for making a figure-of-eight loop. Moreover, the reduction and fixation of fracture were achieved simultaneously by tightening the tension band wire. We confirmed the fixation was stable with only tension band wiring through a full range of knee joint motion. This reinforced technique of tension band wiring was firm enough for postoperative rehabilitation to be carried on after 2 weeks of a long leg cast immobilization after the operation. The most certain advantage of this technique was to be able to reduce anatomically and fixate firmly with appropriate compression simultaneously at the fracture site, where tensile forces are exerted from the avulsing patellar tendon.

Considering that most patients with tibial tuberosity fractures are elite adolescent athletes, the early recovery of the previous activity level is critical in their rehabilitation. A long period of cast immobilization of the knee joint may result in joint stiffness and quadriceps wasting, which would further delay the return to sports. Pretell-Mazzini *et al*.^9^ reported that most (98%) of the patients achieved full range of knee motion at a mean of 22.3 weeks postoperatively, and 94% of the patients were able to resume sports activity at the same level as before the injury at a mean of 28.9 weeks postoperatively in their systematic review. However, Nikiforidis *et al*.^7^ reported that a full range of motion was achieved between 4 and 8 weeks postoperatively, and resume sports activity at the same level as before the injury at a mean of 3 months postoperatively. In this study, the patient achieved the full range of motion at 6 weeks postoperatively and resumed sports activity at the same level as before the injury at 2 months postoperatively. The tension band wiring is more effective in stabilizing the fracture, allowing early joint motion, and avoiding long periods of cast immobilization.

There are some limitations to this surgical technique. In the setting of one case, it is not possible to conclude the superiority of this modified technique over the established tension band wiring technique. A large case series is necessary for qualifying this modified technique. There are various surgical techniques for tibial tuberosity fracture depending on the size and comminution of the fragment. Future studies should be established using the prospective randomized control trial to confirm the effect of this modification technique according to the size and comminution of the fragment.

## Conclusions

We recommend open reduction internal fixation with the reinforced technique of tension band wiring for displaced tibial tuberosity fractures in adolescent athletes. With this reinforced technique, the reduction and fixation of fractures were achieved simultaneously by tightening the tension band wire. Moreover, rigid fixation and early rehabilitation allow young athletes to resume their previous sports activities as soon as possible.

## Ethical approval

This case report is exempt from ethical approval.

## Consent

Informed consent was obtained from the patient.

## Sources of funding

Not applicable.

## Authors contribution

C.L. and S.J.Y.: data collection and writing the paper; Y.J.: data collection; Y.-G.P.: final correction and approval.

## Conflicts of interest disclosure

There are no conflicts of interest.

## Research registration unique identifying number (UIN)

1. Name of the registry: not applicable.
2. Unique identifying number or registration ID: not applicable.
3. Hyperlink to your specific registration (must be publicly accessible and will be checked): not applicable.

## Guarantor

Chaemoon Lim (corresponding author) is the guarantor of this study.

## Data availability statement

The current study is publicly available.

## Provenance and peer review

Not commissioned, externally peer-reviewed.
